# Empirically Tested Health Literacy Frameworks

**DOI:** 10.3928/24748307-20191025-01

**Published:** 2020-02-11

**Authors:** Joycelyn Cudjoe, Sabianca Delva, Mia Cajita, Hae-Ra Han

## Abstract

**Background:**

Health literacy is a significant determinant of health behaviors, but the pathways through which health literacy influences health behaviors are not completely clear nor consistent. The purpose of this systematic review is to critically appraise studies that have empirically tested the potential pathways linking health literacy to health behavior.

**Methods:**

We performed searches of the electronic databases PubMed, Embase, and CINAHL to identify studies that proposed a conceptual framework and empirically tested the proposed mechanism through which health literacy influences certain health behaviors. Twenty eligible studies were included for analysis.

**Key Results:**

The 20 studies addressed various health behaviors: chronic disease self-management (*n* = 8), medication adherence (*n* = 2), overall health status (*n* = 4), oral care (*n* = 1), cancer screening (*n* = 1), shared decision-making (*n* = 1), health information sharing (*n* = 1), physical activity and eating behaviors (*n* = 1), and emergency department visits (*n* = 1). Most studies were conducted in the United States (*n* = 13) and used a cross-sectional design (*n* = 15). The Short Test of Functional Health Literacy in Adults was commonly used to assess health literacy levels. Selection of variables and their operationalization were informed by a theoretical model in 12 studies. Age, gender, race/ethnicity, and insurance status were reported antecedents to health literacy. The most commonly tested mediators were self-efficacy (*n* = 8) and disease knowledge (*n* = 4). Fit indices reported in the studies ranged from acceptable to excellent.

**Discussion:**

Current evidence supports self-efficacy as a mediator between health literacy and health behavior. Further research is needed to identify how health literacy interplays with known psychosocial factors to inform people's use of preventive care services. Future studies should include more disadvantaged populations such as immigrants with high disease burden and those with low health literacy. Theory-based, empirically tested health literacy models can serve as the conceptual basis for developing effective health interventions to improve health behaviors and ultimately decrease the burden of disease in such vulnerable populations. **[*HLRP: Health Literacy Research and Practice*. 2020;4(1):e21–e44.]**

**Plain Language Summary:**

This review systemically compiles, and critically appraises 20 existing studies that test conceptual frameworks that propose potential pathways through which health literacy affects health behaviors. The findings from this review can help inform the development of health literacy-focused interventions to improve the health behaviors of populations with disease burdens.

Health literacy (HL) is a multidimensional concept that addresses a range of skills people need to effectively and efficiently function in a health care environment (Baker, 2006; Guzys, Kenny, Dickson-Swift, & Threlkeld, 2015; Kindig, Panzer, & Nielsen-Bohlman, 2004). People of older age and those who belong to low-income, low-education, immigrant, and ethnic/racial minority groups often have low HL levels and have been found to have poor health outcomes (Crook, Stephens, Pastorek, Mackert, & Donovan, 2016; Diviani, van den Putte, Giani, & van Weert, 2015; Feinberg, Greenberg, & Frijters, 2015).

There has been a proliferation of studies on the impact of HL on health behavior (e.g., self-care, chronic disease management) and overall health outcomes (Guzys et al., 2015; Kim & Han, 2016; Oldach & Katz, 2014). These studies discuss the direct relationship between HL and health behaviors or health outcomes at the bivariate level. Recently, a growing body of research has revealed comprehensive pathways related to HL and health behaviors or outcomes. For example, psychosocial factors such as disease knowledge, self-efficacy, and decisional balance, which are known determinants of health behaviors, were affected by HL levels, and some studies have identified these psychosocial factors as potential mediators to the relationship between HL and health behavior (Harvey, Vegesna, Mass, Clarke, & Skoufalos, 2014; Hui et al., 2014; Kaufman, Mirkovic, & Chan, 2017; Kim & Han, 2016; Oldach & Katz, 2014; Tanaka, Strong, Lee, & Juon, 2013). However, what remains unclear is how theory informs the development of HL conceptual frameworks and the methods used to empirically assess the proposed pathways through which HL influences health behavior (Alper, 2018; Kim & Han, 2016; Oldach & Katz, 2014; Sørensen et al., 2012).

It is important to gain a comprehensive understanding of the theories that guide the systematic application and evaluation of variables used in addressing HL and health behaviors (Alper, 2018). The purpose of this systematic review is to critically appraise studies that tested a theory-based HL conceptual framework. In addition, we were interested in discussing mechanisms through which HL influences health behavior and/or health outcome to build on empirical evidence.

## Methods

## Search Strategy

In October 2017 we performed searches on the electronic databases PubMed, Embase, and CINAHL to find studies that identify and empirically test a HL conceptual framework. Searches were not limited to a specific year. With the assistance of a health science librarian, we identified and used the following keywords and medical subject headings in searching the electronic databases for relevant studies: “health literacy,” “theoretical models,” and “conceptual frameworks” (see **Table A** for specific search terms that were used). Search terms were also truncated and exploded (i.e., search terms were used to retrieved all references indexed to that term), and other relevant Boolean operators were used to make the search as sensitive as possible. Electronic searches were also supplemented by a search on Google Scholar, and the reference lists of relevant articles were examined for articles that were not indexed by the electronic databases. In March 2019, we performed an additional database search using the same strategies we used in the initial search.

### Study Eligibility

All studies were analyzed for their relevance for the purpose of our review. Studies that addressed the impact of HL on a health behavior or health outcome, described and empirically tested a conceptual framework, and were written in English were included in this review. Studies were excluded if they addressed HL as a study concept but did not empirically test a conceptual framework, did not address the impact of HL on health behavior, and were not published in English. Case studies, qualitative studies, conference abstracts, and study protocols and non–peer-reviewed editorial works were also excluded. For the purposes of this article, we define conceptual framework as a product that “graphically or narratively explains study variables and the presumed relationships among them” (Maxwell, 2013).

### Study Selection and Data Extraction

**Figure 1.**
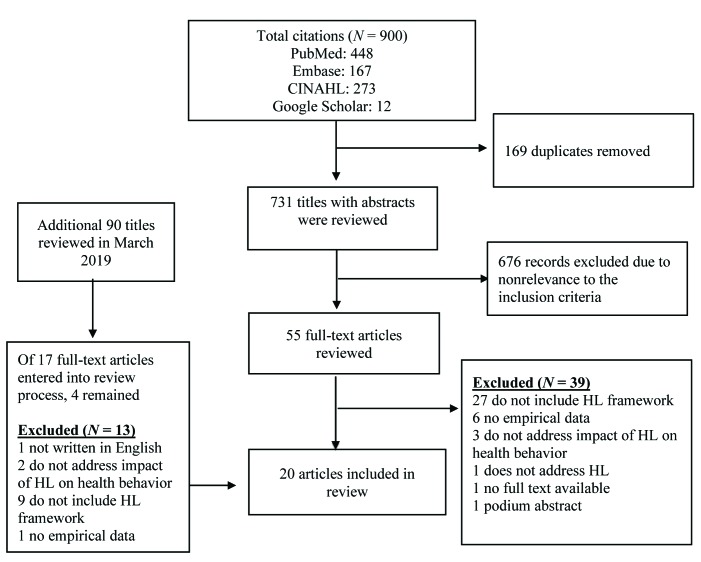
Study selection process. HL = health literacy.

Covidence, an Internet-based software platform that streamlines the production of systematic reviews, was used in the study selection and data extraction process. Our initial database search yielded a total of 900 studies, of which 169 duplicates were removed. To enhance the rigor of the systematic review process, two authors (J.C. and S.D.) independently screened all abstracts and titles for relevance to empirical testing of HL models and frameworks. All conflicts and discrepancies were discussed and resolved through face-to-face group discussions. A total of 676 articles were excluded for nonrelevance to our study's purpose. The full texts of 55 relevant abstracts were then reviewed independently by the study authors (J.C., S.D., M.C., and H.H.) using the study's inclusion and exclusion criteria. We excluded 39 studies for the following reasons: (a) studies did not include or propose an HL framework (*n* = 27); (b) no empirical data were presented (*n* = 6); (c) studies did not address the impact of HL on health behavior (*n* = 3); (d) studies do not include HL as a study variable (*n* = 1), (e) no full text was available (*n* = 1); and (f) it was a podium presentation (*n* = 1). Using the same search terms (**Table A**), an additional database search was conducted in March 2019 for studies published since November 2018. After removing duplicates, 90 titles with abstracts were reviewed for relevance. Two study authors (J.C. and S.D.) independently reviewed 17 full texts using the study's inclusion and exclusion criteria. A total of 13 articles were excluded for the following reasons: (a) studies did not propose a HL framework (*n* = 9); (b); studies did not address the impact of HL on health behavior (*n* = 2); (c) studies were not written in English (*n* = 1); and (d) no empirical data were presented (*n* = 1). **Figure 1** provides a detailed description of the selection process. Two study authors (J.C. and S.D.) extracted data from a total of 20 studies for this systematic review. To enhance interrater reliability and the accuracy of information presented, the authors compared key findings and other relevant data, and discrepancies were resolved.

### Quality Assessment

The Joanna Briggs Checklist was the appraisal tool used in the quality assessment of all studies included in this review (Joanna Briggs Institute, 2018). The checklist is a series of questions that authors of observational studies are expected to answer to enhance a study's methodological rigor. Specifically, each study's quality was assessed using seven items addressing selection bias, measurement bias, confounding variables, and appropriate use of statistical analyses (Joanna Briggs Institute, 2018). Studies were assigned a score of 1 for items that were adequately described, and a score of 0 for items that were not addressed by the authors. Total scores for each study ranged from 0 to 7, with a higher total score attributed to higher quality rating. Studies with a total score less than 3 were rated as low quality, studies with total scores ranging from 3 to 4 were rated as medium quality, and studies with total scores of 5 or higher were rated as high quality. Findings from the quality assessments were used to critique the overall methodological strengths and weaknesses of the studies

Results of the quality assessment process are shown in **Table 1**. All of the studies adequately described inclusion criteria and the characteristics of study participants. There was adequate discussion of items addressing selection bias in most studies included in the review: description of inclusion criteria (*n* = 19), and description of study characteristics (*n* = 15). Most studies included in the review inadequately addressed measurement bias: identification of confounders (*n* = 8), use of valid and reliable measurement of outcome (*n* = 6), and strategy addressing confounders (*n* = 8). The measurement of outcomes in more than 75% (*n* = 15) of studies was based on self-reports. Overall, most studies had high (*n* = 10) to medium (*n* = 6) quality ratings. Only four studies received a low-quality rating.

## Results

### Overview of Studies Included

The characteristics of all 20 studies included in this review are detailed in **Table 2**. Most of the studies were published in the United States (*n* = 13) (Brega et al., 2012; Chen, 2014; Cho, Lee, Arozullah, & Crittenden, 2008; Como, 2018; Crook et al., 2016; Guo et al., 2014; Hickman, Clochesy, & Alaamri, 2016; Jin, Lee, & Dia, 2019; Osborn, Cavanaugh, et al., 2011; Osborn, Cavanaugh, Wallston, & Rothman, 2010; Osborn, Paasche-Orlow, Bailey, & Wolf, 2011; Schillinger, Barton, Karter, Wang, & Adler, 2006; Soones et al., 2017), with the remaining studies published in China (*n* = 2) (Sun et al., 2013; Zou, Chen, Fang, Zhang, & Fan, 2017), Taiwan (*n* = 2) (Hou et al., 2018; Y. J. Lee et al., 2016), Thailand (*n* = 2) (Intarakamhang & Intarakamhang, 2017; Photharos, Wacharasin, & Duongpaeng, 2018), and South Korea (*n* = 1) (E. H. Lee, Lee, & Moon, 2016). Study designs included cross-sectional (*n* = 19) (Brega et al., 2012; Chen, 2014; Cho et al., 2008; Como, 2018; Crook et al., 2016; Guo et al., 2014; Hickman et al., 2016; Hou et al., 2018; Jin et al., 2019; E. H. Lee et al., 2016; Y. J. Lee et al., 2016; Osborn, Cavanaugh, et al., 2011; Osborn et al., 2010; Osborn, Paasche-Orlow, et al., 2011; Photharos et al., 2018; Schillinger et al., 2006; Soones et al., 2017; Sun et al., 2013; Zou et al., 2017) and mixed methods (*n* = 1) (Intarakamhang & Intarakamhang, 2017). Sample sizes ranged from 62 to 2,594, with only seven studies calculating sample sizes *a priori* (Chen, 2014; Como, 2018; Hou et al., 2018; Intarakamhang & Intarakamhang, 2017; E. H. Lee et al., 2016; Y. J. Lee et al., 2016; Photharos et al., 2018).

Study participants in all the U.S.-based studies were predominately female, urban dwellers, adults (age range, 18–75 years) with less than a high school education. In addition, the samples in U.S.-based studies were more than 50% ethnic/racial minority groups (i.e., Black, Hispanic, Native American/Alaska Native) except for three studies that included more than 60% White participants (Chen, 2014; Guo et al., 2014; Osborn, Cavanaugh, et al., 2011). One U.S.-based study (Crook et al., 2016), however, did not report the race or ethnicity of study participants. All studies in this systematic review included adult participants (age >18 years) except for one study in Thailand that used national data from school-age children between ages 9 and 14 years (Intarakamhang & Intarakamhang, 2017).

All studies measured one or more subdimensions of HL. Eight studies measured print literacy (Brega et al., 2012; Chen, 2014; Cho et al., 2008; Como, 2018; Jin et al., 2019; Osborn, Cavanaugh, et al., 2011; Osborn et al., 2010; Sun et al., 2013), four studies measured numeracy (Brega et al., 2012; Como, 2018; Crook et al., 2016; Soones et al., 2017), and four studies measured functional literacy (Hou et al., 2018; Osborn, Paasche-Orlow, et al., 2011; Photharos et al., 2018; Schillinger et al., 2006). Three studies addressed disease-specific HL: diabetes (Osborn, Cavanaugh, et al., 2011; Osborn et al., 2010) and heart failure (Zou et al., 2017). All studies used an existing and well-validated HL measure except one study in Thailand that developed and validated the Health Literacy Scale for Thai overweight children (Chronbach's alpha: 0.70) (Intarakamhang & Intarakamhang, 2017). The most common HL measures were the Rapid Estimate of Adult Literacy in Medicine (REALM) (Osborn, Cavanaugh, et al., 2011; Osborn et al., 2010), Short Test of Functional Health Literacy in Adults (S-TOFHLA) (Cho et al., 2008; Como, 2018; Soones et al., 2017), and Test of Functional Health Literacy in Adults (TOFHLA) (Osborn, Paasche-Orlow, et al., 2011; Schillinger et al., 2006). Additional measures included the Health Literacy Scale, Brief Health Literacy Tool, the Mandarin version of the European Health Literacy Survey Questionnaire, and the Chinese Version of Health Literacy Scale for Patients with Chronic Disease (E. H. Lee et al., 2016; Y. J. Lee et al., 2016; Zou et al., 2017), which were mostly used in international studies (Taiwan, South Korea, Thailand, and China) to assess functional HL in the context of breast cancer, chronic kidney disease, diabetes, and heart failure management. Similarly, two studies (Guo et al., 2014; Hickman et al., 2016) conducted in the U.S. across ethnically diverse samples (predominantly Black, non-Hispanic middle-aged women) assessed functional literacy using Chew's 3-item scale and 1-item scale (Chew et al., 2008).

### Antecedents and Outcomes of HL

**Table 3** details the antecedents, mediators, moderators, and outcomes of HL as outlined in the studies. All but four studies identified demographics and psychosocial factors as the most common antecedent to HL (Hickman et al., 2016; Osborn et al., 2010; Photharos et al., 2018; Zou et al., 2017). The authors reported the following sociodemographic and medical characteristics: age, education, income, health insurance status, race/ethnicity (Brega et al., 2012; Chen, 2014; Cho et al., 2008; Como, 2018; Guo et al., 2014; Hou et al., 2018; Osborn, Paasche-Orlow, et al., 2011; Schillinger et al., 2006), general literacy and language (English proficiency) (Schillinger et al., 2006), marital status (Como, 2018; Y. J. Lee et al., 2016), Internet use (Crook et al., 2016; Jin et al., 2019), disease duration (Y. J. Lee et al., 2016), and cognition (Soones et al., 2017). Older age (Hou et al., 2018; Osborn, Paasche-Orlow, et al., 2011), low education (Osborn, Paasche-Orlow, et al., 2011), and Black race (Osborn, Cavanaugh, et al., 2011; Osborn, Paasche-Orlow, et al., 2011) were linked to low HL, whereas increased years of education (Schillinger et al., 2006; Sun et al., 2013) and Internet use (Crook et al., 2016; Jin et al., 2019) were linked to high HL; however, a study conducted in China with a sample of older adults with low-income (*N* = 295, mean age of 58 years) reported no association between age and HL (Y. J. Lee et al., 2016). Psychosocial antecedents included perceived health knowledge and perceived knowledge (Crook et al., 2016; Y. J. Lee et al., 2016; Sun et al., 2013). A statistically significant association was reported among perceived empowerment, prior knowledge, and HL (Y. J. Lee et al., 2016; Sun et al., 2013). One study among a sample of predominantly middle-aged (mean age, 38 years) women (69%) reported a nonstatistically significant association between perceived heart health knowledge and HL (Crook et al., 2016). The lack of association can be attributed to potential selection bias.

Studies addressed the following health behaviors and health outcomes: chronic disease self-management (*n* = 9) (Brega et al., 2012; Chen, 2014; Hickman et al., 2016; Y. J. Lee et al., 2016; Osborn et al., 2010; Osborn, Paasche-Orlow, et al., 2011; Photharos et al., 2018; Schillinger et al., 2006; Zou et al., 2017), colorectal cancer screening (*n* = 1) (Jin et al., 2019), medication adherence (n = 2) (Osborn, Cavanaugh, et al., 2011; Soones et al., 2017), overall health status (*n* = 4) (Como, 2018; Hou et al., 2018; E. H. Lee et al., 2016; Sun et al., 2013), oral care (*n* = 1) (Guo et al., 2014), health information sharing (*n* = 1) (Crook et al., 2016), physical activity and eating behaviors (*n* = 1) (Intarakamhang & Intarakamhang, 2017), shared decision-making in relation to breast cancer care (*n* = 1) (Hou et al., 2018), and emergency department visits (*n* = 1) (Cho et al., 2008). These studies reported that HL leads to better self-care and medication adherence, improved health status, improved self-reported oral health, less frequent emergency department visits, shorter hospitalizations, and improved physical activity and healthy eating behaviors (Brega et al., 2012; Cho et al., 2008; Guo et al., 2014; Hou et al., 2018; Intarakamhang & Intarakamhang, 2017; Soones et al., 2017; Sun et al., 2013; Zou et al., 2017). However, HL did not affect information-sharing behaviors (Crook et al., 2016), patients' participation in shared decision-making (Hou et al., 2018), and colorectal cancer screening (Jin et al., 2019). Six studies did not find a significant association between HL and reported health behaviors (physical activity, medication adherence, glycemic control) or health outcomes (self-rated health of patients with diabetes and chronic heart failure) (Como, 2018; Y. J. Lee et al., 2016; Osborn, Cavanaugh, et al., 2011; Osborn et al., 2010; Osborn, Paasche-Orlow, et al., 2011; Schillinger et al., 2006).

### Pathways Linking HL and Health Behaviors/Outcomes

All but three studies assessed a number of variables as possible mediators between HL and health behaviors/outcomes (Hou et al., 2018; Intarakamhang & Intarakamhang, 2017; Schillinger et al., 2006). Eight studies examined the mediating effect of self-efficacy on the relationship between HL and diabetes management, heart failure management, and general self-care (Como, 2018; Chen, 2014; E. H. Lee et al., 2016; Y. J. Lee et al., 2016; Osborn et al., 2010; Osborn, Paasche-Orlow et al., 2011; Photharos et al., 2018; Zou et al., 2017). Of the five studies that measured disease-specific (diabetes, heart failure, chronic kidney disease) self-efficacy (E. H. Lee et al., 2016; Y. J. Lee et al., 2016; Osborn et al., 2010; Photharos et al., 2018; Zou et al., 2017), four studies found self-efficacy as a statistically significant mediator (E. H. Lee et al., 2016; Y. J. Lee et al., 2016; Osborn et al., 2010; Zou et al., 2017). However, only two studies (E. H. Lee et al., 2016; Y. J. Lee et al., 2016) controlled for possible demographic confounders (age, gender, education, marital status).

Four studies that examined how HL is related to health behavior through disease knowledge found the following: only one study showed a statistically significant mediating effect of knowledge in the context of diabetes management (Brega et al., 2012), and three studies found a direct association between HL and knowledge (Chen, 2015; Cho et al., 2008; Osborn, Paasche-Orlow et al., 2011). All four studies that examined the mediating effect of disease knowledge did not describe how knowledge instruments were scored, however. In addition, all four studies had a large proportion (65%–70%) of study participants with a high school education or less (Chen, 2015; Cho et al., 2008; Osborn, Paasche-Orlow et al., 2011; Zou et al., 2017).

Of the eight studies that examined self-care activities (medication adherence, physical activity, self-monitoring of blood glucose, foot care, healthy diet) as factors linking the pathway between HL and health outcomes (glycemic control, emergency department visits, blood pressure control, and physical and mental health status) (Brega et al., 2012; Cho et al., 2008; Como, 2018; Hickman et al., 2016; E. H. Lee et al., 2016; Y. J. Lee et al., 2016; Osborn, Paasche-Orlow, et al., 2011; Sun et al., 2013), two reported a significant, mediating effect (Brega et al., 2012; E. H. Lee et al., 2016). Both studies controlled for known demographic covariates such as age, gender, education, marital status, treatment regimen (insulin or oral hypoglycemic use), hemoglobin A1c level, as well as duration of disease in the mediation analysis (Brega et al., 2012; E. H. Lee et al., 2016).

Other proposed mediators included patient-provider interaction (Guo et al., 2014; Hickman et al., 2016), decisional balance (Como, 2018), medication compliance (Cho et al., 2008; Soones et al., 2017), preventive care use (Cho et al., 2008; Guo et al., 2014), information overload (Como, 2018) and attitude and beliefs toward information (Crook et al., 2016). Only one study across a sample of predominately White (66%), urban-dwelling adults (mean age, 53 years) found that patient-dentist communication and the frequent use of dental care services mediates the relationship between HL (navigation) and self-rated oral health (*p* = .01) (Guo et al., 2014). The remaining studies found no statistically significant mediation pathways linking HL to health behaviors and outcomes (Cho et al., 2008; Crook et al., 2016; Hickman et al., 2016; Soones et al., 2017). Only 3 of the 20 studies included in this review assessed the interaction of HL and study outcomes (glycemic control, medication adherence), but the authors did not describe this relationship as moderating (Osborn, Paasche-Orlow et al., 2011; Schillinger et al., 2006; Soones et al., 2017).

### Validation of Theory-Based Conceptual Frameworks

Fourteen studies (Chen, 2014; Crook et al., 2016; Guo et al., 2014; Hickman et al., 2016; Hou et al., 2018; Intarakamhang & Intarakamhang, 2017; E. H. Lee et al., 2016; Y. J. Lee et al., 2016; Osborn, Cavanaugh, et al., 2011; Osborn, Cavanaugh et al., 2010; Osborn, Paasche-Orlow et al., 2011; Schillinger et al., 2006; Sun et al., 2013; Zou et al., 2017) reported good to excellent goodness of fit in which all indices were statistically significant; two studies did not report fit indices (Como, 2018; Jin et al., 2019). Of the 20 studies included in this review, all but one hypothesized the relationships among proposed study variables (E. H. Lee et al., 2016). Twelve studies used theory to inform the selection and operationalization of study variables (Chen, 2014; Como, 2018; Crook et al., 2016; Hickman et al., 2016; Hou et al., 2018; Intarakamhang & Intarakamhang, 2017; Jin et al., 2019; Y. J. Lee et al., 2016; Osborn, Paasche-Orlow, et al., 2011; Photharos et al., 2018; Sun et al., 2013; Zou et al., 2017). Three studies validated the theory by Paasche-Orlow and Wolf (2007) across a sample of low-income, middle-aged (>50 years) adults with chronic disease (Como, 2018; Y. J. Lee et al., 2016; Osborn, Paasche-Orlow, et al., 2011). Of the three studies, one study (Y. J. Lee et al., 2016), which used participants' self-reports of glycemic control, showed an acceptable framework fit, and an excellent framework fit was reported for the study (Osborn, Paasche-Orlow, et al., 2011) that used patients' medical records. One study validated the Nutbeam HL model (Nutbeam, 2008) in the context of obesity prevention using a national sample of school-age children (*N* = 2,000; age range, 9–14 years); fit indices indicated a good fit (Intarakamhang & Intarakamhang, 2017). One study conducted in China with a sample of city-dwelling adults (*N* = 3,222) validated an adapted framework of various HL theoretical models (Baker [2006], Paasche-Orlow and Wolf [2007], and McCormack [2009] models) and reported a good fit of the proposed framework (Sun et al., 2013). The authors of the study did not clearly describe how study variables were operationalized, however (Sun et al., 2013). Two studies conducted in the U.S. (Como, 2018; Jin et al., 2019) also adapted multiple theoretical models (i.e. Paasche-Orlow and Wolf model [2007], Bandura's self-efficacy theory [Bandura, 1977], health literacy skills framework [Squires, Peinado, Berkman, Boudewyns, & McCormack, 2012] and cognitive mediation model [Eveland & Dunwoody, 2001]) but failed to report fit indices. Additionally, five studies (Chen, 2014; Crook et al., 2016; Hickman et al., 2016; Photharos et al., 2018; Zou et al., 2017) that reported good to excellent fit indices were informed by theories that do not specifically address HL but are commonly used in nursing and public health research to study health behaviors and overall health outcomes: Orem's theory of self-care and Bandura's social cognitive theory, theory of diffusion of innovations, model of client health behavior, individual and family self-management theory, and capability opportunity motivation and behavior model. (Bandura, 1977; Cox, 1982; Michie, Stralen, van Stralen, & West, 2011; Orem, 2003; Rogers, 2002; Ryan & Sawin, 2009.)

## Discussion

To our knowledge, this is the first systematic review to critically appraise studies that have empirically tested the potential pathways linking HL to health behaviors and health outcomes. We found evidence to support that theoretically selected mediators (i.e., self-efficacy, disease knowledge, self-care activities, and patient-provider communication) mediate the identified relationship between HL and chronic disease management, with self-efficacy as the commonly tested mediator (E. H. Lee et al., 2016; Y. J. Lee et al., 2016). Our findings show that unless people possess adequate HL, they may perceive low confidence in their abilities to manage their chronic diseases. In addition, improving people's HL is an essential first step to increasing their knowledge about their disease, improving their ability to adequately perform self-care activities, and effectively communicate and collaborate with health care providers in their chronic disease management (Charlot et al., 2017; Chisholm-Burns, Spivey, & Pickett, 2018). We also found evidence to support that intervention outcomes (glycemic control, medication adherence) differ by the HL levels of study participants, suggesting HL as a moderator (Schillinger et al., 2006; Soones et al., 2017). This finding highlights an important implication for future research, particularly in relation to intervention research as it relates to the role of HL beyond mediation.

We identified several factors that may have contributed to the mixed findings we reported: study design, selection bias, small sample sizes, measurement errors, and non–theory-guided operationalization of study variables. Although all studies in this review aimed to examine the pathways linking HL to health behaviors and outcomes, these studies exclusively used cross-sectional and a mixed-methods designs, which preclude causality and temporality. Secondly, only 7 of 20 studies conducted sample size calculations and power analyses *a priori* (Chen, 2015; Como, 2018; Hou et al., 2018; Intarakamhang & Intarakamhang, 2017; E. H. Lee et al., 2016; Y. J. Lee et al., 2016; Photharos et al., 2018). The lack of statistical power in most of the studies could account for the mixed findings reported. Thirdly, although all U.S.-based studies used well-validated HL measures, the remaining studies either lacked psychometric testing results or had only been tested in a single population; therefore, the validity and reliability of those measures could not be established (Intarakamhang & Intarakamhang, 2017; E. H. Lee et al., 2016; Y. J. Lee et al., 2016; Sun et al., 2013; Zou et al., 2017). Also important is that the studies were predominantly across a convenience sample of female, urban-dwelling adults with less than a high school education who were recruited from health care facilities. Therefore, findings cannot be generalized to other populations that do not use the health care system due to language barriers or a lack of health insurance. Finally, theory provides a systematic foundation and a logical pathway for illustrating the relationship among various study concepts and variables. However, only a limited number of studies (*n* = 12) included in the review explained how theory informed the selection and operationalization of study variables, delimiting the generalizability of findings.

Findings from this review call for the need to use theoretically grounded, methodologically rigorous research with statistically powered sample sizes to adequately examine the interplay between HL and health behaviors or outcomes in diverse study populations. For example, the studies included in this review exclusively used a cross-sectional design to test the indirect pathways linking HL to health behaviors. Hence, there is still a need for establishing temporality and causality using more rigorous study designs such as longitudinal cohort design. Several studies have used longitudinal data to examine the role of HL on health behaviors and outcomes; however, they did not meet the inclusion criteria for this review because the authors did not specify a HL conceptual framework to be tested (Kobayashi, Wardle, & Wagner, 2015; Washington, Curtis, Waite, Wolf, & Paasche-Orlow, 2018). In addition, although a recent systematic review showed that HL has gained importance on the European health agenda, none of the studies identified from our extensive search of various database were conducted in Europe (Sørensen et al., 2015). Further, among U.S.-based studies, all were conducted on female, English-speaking adults (Brega et al., 2012; Chen, 2014; Cho et al., 2008; Como, 2018; Crook et al., 2016; Guo et al., 2014; Hickman et al., 2016; Jin et al., 2019; Osborn, Cavanaugh, et al., 2011; Osborn et al., 2010; Osborn, Paasche-Orlow, et al., 2011; Schillinger et al., 2006; Soones et al., 2017). Although people who belong to ethnic/racial minority groups and those with low English proficiency, particularly immigrants, are known to be disproportionately burdened by low HL, they were excluded from the U.S.-based studies (Alper, 2018; Wang et al., 2013). In particular, African immigrants, an exponentially increasing immigrant group in the U.S. with worse health outcomes in comparison to other immigrant groups, were excluded in all the U.S.-based studies (Anderson, 2015). Although there is a possibility that African immigrants were categorized as Black Americans in some of these studies, it has been established that people of African descent (Black, African immigrant, and Afro-Caribbean) in the U.S. have different cultural and linguistic characteristics that affect their health outcomes differently. Therefore, there is a need to disaggregate these subgroups in health research (Commodore-Mensah et al., 2017; Forney-Gorman & Kozhimannil, 2016).

## Study Stregnths

The Cochrane Collaboration and the U.S. Institute of Medicine have endorsed that review teams must have content and methodological expertise (Bigendako & Syriani, 2018; Gøtzsche & Ioannidis, 2012; Institute of Medicine, 2011). A major strength of this study is that our contributors have undergone training in systematic review methodology and have published prior reviews (Cajita, Cajita, & Han, 2016; Han, Floyd, et al., 2018; Han, Kim, et al., 2018). Additionally, most of the authors are clinicians with expertise in health promotion among populations with poor health literacy. These skill-sets helped us capture a heterogeneity of opinions and allowed for high interrater reliability when reviewing articles for inclusion in the review. These strengths add to the degree of confidence when reporting our study findings, which also speaks to the thoroughness of this systematic review.

## Study Limitations

This systematic review is limited in that despite our extensive database searches, there may be other relevant and unpublished studies that may not have been identified. Therefore, the theories we identified as guiding the development of HL conceptual frameworks may not be exhaustive. The majority of studies included in this review assessed HL using REALM and TOFHLA, which assess reading ability and comprehension, respectively, but do not comprehensively address the multidimensionality of HL (i.e., ability to understand written text, speak and listen effectively, and use quantitative data to make appropriate health decisions) (Sørensen et al., 2012). Most studies used a cross-sectional design that precludes causality and temporality. In addition, we only included studies published in English. This may have also resulted in the small number of studies included in this review as well as the number of studies that included non–English-speaking populations.

## Conclusion

Our review adds to the existing body of knowledge on the impact of HL on health behavior by providing a comprehensive understanding of how theory informs the development of HL conceptual frameworks, and the systematic selection and evaluation of variables that inform HL-focused studies. We found evidence to support that HL is related to health behaviors, particularly chronic disease management, through mediators such as self-efficacy and disease knowledge.

## Figures and Tables

**Table 1:** Quality Assessments of Studies

**Reference**	**Description of Inclusion Criteria**	**Description of Study Characteristic**	**Standard Criteria Used for Measurement of the Condition**	**Identification of Confounders**	**Strategies for Addressing Confounding Factors**	**Valid and Reliable Measurement of Outcome**	**Statistical Analyses**	**Overall Quality**
Brega et al. (2012)	1	1	1	1	1	1	1	High
Chen et al. (2014)	1	1	1	0	0	0	1	Medium
Cho, Lee, Arozullah, & Crittenden (2008)	1	1	0	0	0	0	1	Medium
Como (2018)	1	1	1	1	1	1	0	High
Crook, Stephens, Pastorek, Mackert, & Donovan (2016)	1	0	0	0	0	0	1	Low
Hou et al. (2014)	1	0	0	1	1	0	1	Medium
Hickman, Clochesy, & Alaamri (2016)	1	1	1	0	0	0	0	Medium
Huo et al. (2018)	1	1	1	0	0	1	1	High
Intarakamhang & Intarakamhang (2017)	1	0	0	0	0	0	1	Low
Jin, Lee, & Dia (2019)	1	1	0	1	1	0	1	High
E.H. Lee, Lee, & Moon (2016)	1	1	0	0	0	0	1	Medium
Y.J. Lee et al. (2016)	1	1	1	0	0	1	1	High
Osborn, Cavanaugh, et al. (2011)	0	0	0	0	0	0	1	Low
Osborn, Cavanaugh, Wallston, & Rothman (2010)	1	1	1	0	0	1	1	High
Osborn, Paasche-Orlow, Bailey, & Wolf (2011)	1	1	0	0	0	0	1	Medium
Photharos, Wacharasin, & Duongpaeng (2018)	1	0	1	0	0	0	0	Low
Schillinger, Barton, Karter, Wang, & Adler (2006)	1	1	1	1	1	1	1	High
Soones et al. (2017)	1	1	1	1	1	0	0	High
Sun et al. (2013)	1	1	0	1	1	0	1	High
Zou, Chen, Fang, Zhang, & Fan (2017)	1	1	0	1	1	0	1	High

Note. 1 = clearly discussed; 0 = not discussed.

**Table 2:** Study Characteristics and Main Findings

**Reference**	**Study Purpose**	**Setting/Sample**	**HL Domains (HL Measure)**	**Main Results**
Brega et al. (2012)	To develop a theoretical framework and test the mechanisms through which HL is associated with outcomes, focusing on the relationship between HL and glycemic control among Native Americans and Alaska Natives with diabetes	2,594 rural-dwelling adults with diabetes Country: United States Age: 18–65 y; Income: <$10,000; 93% less than college graduates Ethnicity: 100% Native American and Alaska Native HL levels: not stated	Print literacy (TOHFLA) Numeracy (not stated)	High HL associated with decreased HbA1c levels (B = −0.070, *p*< .05). Significant association between high HL and healthy behaviors (frequent healthy diet, monitor blood sugar). Self-monitoring of blood sugar mediates HL and glycemic control (B = −0.028, *p*< .05). Diabetes knowledge is a significant mediator between HL and glycemic control (beta = −0.134, *p*< .05)
Chen et al. (2014)	Test a model to explain the relationships between HL, heart failure knowledge, self-efficacy, and self-care	63 urban-dwelling adults with heart failure Country: United States Mean age: 62.1 y; mean years of education: 13.7 y; female: 47.6% Ethnicity: 86% White; 11% Black, 2% Hispanic/Latino, 2% Native American/Alaska Native HL levels: inadequate 16%, marginal 16%, adequate, 68%	Print literacy (s-TOHFLA)	Direct relationship between HL and heart failure knowledge (beta = 0.46, *p*< .05). Heart failure knowledge and self-efficacy do not mediate the relationship between HL and heart failure self-care
Cho, Lee, Arozullah, & Crittenden (2008)	Explore intermediate factors that link HL to health status and use of health services (ED visit, hospitalization)	489 urban-dwelling adults with Medicare Country: United States Age: >65 y Average education level: HS graduate; female: 78.7% Ethnicity: 59.1% Black HL levels: inadequate 51%	Print literacy/comprehension (s-TOFHLA)	Positive, direct relationships between HL, health status (beta = 0.48, *p*< .05); direct negative relationship between HL and hospitalization and ED visits respectively (beta = −0.24 and beta = −0.35). Compliance and disease knowledge are not significant mediators between HL and outcomes (health status, hospitalizations, ED visit). HL mediates educational attainment and outcomes (health status, hospitalization and ED visits)
Como (2018)	Investigate whether HL, self-efficacy, and medication adherence can explain or predict the variance in health outcomes (perceived physical or mental health status) in persons with chronic heart failure	175 urban-dwelling adults diagnosed with heart failure and attending cardiology health centers in New York, NY Country: United States Mean age: 73 y; male: 66.9% Ethnicity: 11.4% Black, 83.4% White, 4% Hispanic/Latino, 0.6% Asian, 0.6% Native American HL levels: inadequate 38.3%, adequate: 45.7%	Print literacy/comprehension (s-TOFHLA) Numeracy (s-TOFHLA)	Self-efficacy is associated with physical health status (*p*= .002). Education, income, marital status (widow), illness severity indicators (number of medication/days, frequency/day) are significant predictors of physical health status (*p*< .001). No associations between HL, medication adherence, and physical health status. Medication adherence does not mediate the relationship between HL and physical health status. Medication adherence (*p*< .001), numeracy (*p*= .029), and reading comprehension (*p*= .049) are associated with mental health status. Medication adherence does not mediate the relationship between HL and mental health status
Crook, Stephens, Pastorek, Mackert, & Donovan (2016)	Explain the associations among perceived health knowledge, information sharing, attitudes, behaviors, and HL	180 English-speaking adults recruited from a central Texas acute and preventive care center Country: United States Age: 18–75 y; mean age 38.7 y +13.2; female: 69% Education: not reported Ethnicity: not reported HL levels: not stated	Numeracy (Newest Vital Sign)	Internet use positively associated with HL level (beta = 0.55, *p*< .001). Attitude toward information mediates relationship between HL and behavioral intention (*p*< .001) as well as the relationship between HL and information sharing (*p*< .001). No significant association between perceived healthy heart knowledge and HL (beta = 0.14, *p*= .14). High perceived healthy heart knowledge associated with positive attitudes toward health information (beta = 0.13, *p*= .03) and lower perception of information overload (beta = −0.14, *p*= .01)
Guo et al. (2014)	Examine effects of HL, patient-dentist communication, dental care patterns on self-rated oral health status	1,799 rural-dwelling adults in Florida Country: United States Mean age: 52.9 y; HS graduate or lower: 53%; female: 53%; Ethnicity: 34% Black, 66% White HL levels: low 31%, high 69%	Navigation (Chew's 3-Item HL scale)	Significant direct association between HL and self-rated oral health (beta = 0.091, *p*< .001). Patient-dentist communication and dental care patterns mediate the relationship between HL and self-rated oral health (beta = 0.003, *p*= .01)
Hickman, Clochesy, & Alaamri (2016)	Examine predictive associations among HL, quality of the provider interaction, perceived communication skills, and behavioral activation on blood pressure control	109 English-speaking, urban-dwelling adults with hypertension in Northeast Ohio Country: United States Mean age: 52 y (±11); education: not reported; Female: 59%; Income: not reported Ethnicity: 68% Black, 24% White, 5% Hispanic, 3% Multiracial HL levels: not stated	Functional (Chew's 1-item scale)	HL (beta = 0.15, *p*< .10), quality of provider interaction (beta = 0.38, *p*< .01), perceived communication skills (beta = 0.22, *p*< .05) directly associated with behavioral activation. Provider interaction (beta = 0.27, *p*< .001) and behavioral activation (beta = −0.29, *p* < .001) are directly associated with blood pressure control
Hou et al. (2018)	To examine the mechanisms and completeness of the Integrated Model of HL	511 adults diagnosed with breast cancer and attending breast surgery clinics and teaching hospitals Country: Taiwan Mean age: 57.9 y; <HS graduate: 31.7%; Married: 71.6%; residence: 75% urban dwellers; employment: 44% unemployed; average duration of cancer diagnosis: 43 months HL levels: inadequate: 37.5%; adequate: 62.5%	Functional, comprehension (Mandarin version of HLS-EU-Q)	Age and cancer stage are inversely related to HL (*p*< .05). Education (beta = 0.41, *p*< .05), cancer duration (beta = 0.27, *p*< .05) significantly associated with HL Significant associations among patients' participation in shared decision-making (beta = 0.46, *p*< .05), self-rated health status (beta = 0.27, *p*< .05) and HL No associations among marital status, place of residence, occupation, and HL
Intarakamhang & Intarakamhang (2017)	Develop a scale for evaluating HL level of overweight children in Thailand and develop a model of health behavior to prevent obesity	2,000 population-based sample of urban and provincial Thai students Country: Thailand Age: 9–14 y; education: not reported; sex: not reported; income: not reported Ethnicity: 100% Asian HL levels: not stated	Media, functional, navigation (HL scale for overweight Thai childrena)	Direct effect of critical skills (media literacy and making appropriate health-related decision) on obesity preventive behaviors (eating, exercise and emotional behaviors) (beta = 0.55, *p*< .05) Basic intelligence skills (health knowledge, accessing information and services) directly related to interactive skills (communication and managing health conditions) (beta = 0.76, *p*< .05) Direct relationship between interactive skills and critical skills (beta = 0.97, *p*< .05)
Jin, Lee, & Dia (2019)	Examine hypothetical pathways through which online health information-seeking behaviors (using emails to communicate with providers, visit social networking site to read and share medical topics) influence HL, which, in turn, leads to colorectal cancer screening among Korean Americans	433 Korean American adults living in the southeastern United States Country: United States Mean age: 57.6 y, female: 60.8%; family history of cancer: 54.6%; no personal history of cancer: 85.4%; education: not reported HL levels: not stated	Print literacy, comprehension (Brief HL Screening Tool)	Online health information seeking behaviors associated with HL (beta = 0.146, *p*< .001) and information overload (beta = 0.179, *p*< .01) Information overload inversely associated with HL (beta = −0.242, *p*< .001). Decisional balance associated with HL (beta = 0.124, *p*< .05), fecal occult blood test (beta = 0.161, *p*< .05) and sigmoidoscopy uptake (beta = 0.169, *p*< .01) HL not significantly associated with fecal occult blood test, sigmoidoscopy, and colonoscopy uptake HL does not mediate the relationship between online information seeking and colorectal cancer screening
E.H. Lee, Lee, & Moon (2016)	Explore the relationships among HL, self-efficacy, self-care activities, and HRQOL	459 Korean-speaking adults diagnosed with type 2 diabetes, recruited from university hospitals in South Korea between 2014 and 2015 Country: South Korea Age: 20–70 y; mean age 59.6 y (±10.57); female: 60%; less than HS graduate: 32%; income: not reported HL levels: not stated	Functional (communication) (Health Literacy Scale)	Direct effect of HL on self-efficacy (beta = 0.45, *p*< .001) and self-care activities (beta = .209, *p*< .001). Self-efficacy mediates relationship between HL and self-care activities (beta = 0.299, *p*= .005). Self-care activities are directly related to HRQOL (beta = 0.399, *p*< .001). No direct effect of HL on HRQOL. Self-care activities mediate relationship between HL and HRQOL (beta = 0.203, *p*= .002). Self-care activities mediate relationship between self-efficacy and HRQOL (beta = 0.265, *p*= 0.004)
Y.J. Lee et al. (2016)	Validate a hypothesized model exploring the influencing pathways of empowerment perceptions, HL, self-efficacy, and self-care to HbA1c levels among patients with type 2 diabetes	295 person convenience sample of adult patients diagnosed with type 2 diabetes >6 months and attending endocrine outpatient clinics in southern Taiwan Country: Taiwan Age: 20–80 y; mean age: 58.2 y; female: 42%; less than HS graduate: 37.3%; Income: 68% low SES HL levels: not stated	Functional (communication) (Health Literacy Scale)	Nonsignificant association between age and HL, HL and self-care behaviors, empowerment and self-efficacy, empowerment and self-care behaviors. HL mediates relationship between empowerment and self-efficacy (beta = 0.39, *p*< .001). Self-efficacy and HL also mediate the relationship between self-care behaviors and empowerment (beta = 0.26, *p*< .001). Self-care behaviors mediates self-efficacy and glycemic control (beta = –.14; *p*< .05)
Osborn, Cavanaugh, et al. (2011)	Test whether HL and/or numeracy are related to diabetes medication adherence and whether either factor explained racial differences in adherence to diabetes medications	383 English -peaking urban, rural, and suburban dwelling adults living in North Carolina and Tennessee diagnosed with types 1 and 2 diabetes Country: United States Age: 18–85 y; Mean age: 54 y; female: 50%; <HS graduate: 44%; income >$20,000: 56% Ethnicity: 35% Black HL levels: not stated	Diabetes-relatednumeracy (Diabetes Numeracy Test) Print literacy (REALM)	HL does not mediate relationship between Black race and diabetes medication adherence. Direct negative association between Black race and HL (beta = −0.28, *p* < .001). Non-significant association between HL and medication adherence (*p*= .06). Direct association between duration of diabetes and medication adherence (beta = 0.13, *p*< .01)
Osborn, Cavanaugh, Wallston, & Rothman (2010)	Examine the predicted pathway linking HL, numeracy, and diabetes self-efficacy to glycemic control	383 English-speaking urban, rural, and suburban dwelling adults living in North Carolina and Tennessee diagnosed with Types 1 and 2 diabetes Country: United States Age: 18–85 y; mean age: 54 y; female: 50%; >HS education: 56%; income >$20,000: 56% Ethnicity: 35% Black HL levels: not stated	Diabetes-related numeracy (Diabetes Numeracy Test) Print literacy (REALM)	Younger age (*p*< .001), insulin use (*p*< .001), increased duration of diabetes diagnosis (*p*< .01), Black race (*p* < .01) are directly associated with higher HbA1c levels. Greater self-efficacy associated with lower HbA1c levels (*r* = −0.25, *p*< .001). Model accounted for 21% variability in HbA1c. No direct relationship between HL and glycemic control (HbA1c). Self-efficacy mediates relationship between general numeracy and glycemic control (*p* < 0.05)
Osborn, Paasche-Orlow, Bailey, & Wolf (2011)	Validate the Paasche-Orlow and Wolf model examining mechanisms linking HL to physical activity and self-reported health status	330 English-speaking adults with hypertension recruited from clinics across the United States. Country: United States Mean age: 53.6 y; female: 68%; <HS education: 70.7%; unemployed: 66%; uninsured: 44% Ethnicity: 79% Black HL levels: not stated	Functional literacy (s-TOFHLA)	Low education (beta = 0.56, *p*< .001), Black race (beta = 0.51, *p* < .001), older age (beta = 0.36, *p*< .001) directly associated with low HL. High HL associated with high knowledge (beta = 0.22, *p* < .001). Self-efficacy directly related with health status (beta = 0.17, *p* < .01). No association between self-care behavior and health status. Nonsignificant relationship between race and self-efficacy (beta = 0.10). Knowledge mediates relationship between HL and self-efficacy (B = 0.045, *p*< .001)
Photharos, Wacharasin, & Duongpaeng (2018)	Develop and test the causal relationships among family functioning, HL, chronic kidney disease self-efficacy, illness perceptions, social support, and self-management behaviors among persons experiencing early stages of chronic kidney disease	275 adults experiencing early stage chronic kidney disease and receiving medical treatment Country: Thailand 60% male; college educated: <68%; family history of chronic kidney disease: 19%; history of hypertension: 36.7%; history of diabetes and hypertension: 29.5% HL levels: not stated	Functional, communication, critical literacy (Health Literacy Scale)	HL (beta = 0.31, *p*< .0), family functioning (beta = 0.53, *p*< .05) directly associated with chronic kidney disease self-efficacy HL (beta = 0.37, *p*< .05), social support (beta = 0.24, *p*< 0.05) directly associated with self-management behaviors Family functioning is related to self-management behaviors through social support (beta = 0.15, *p*< .05) Chronic kidney disease self-efficacy does not mediate the relationships among HL, family functioning, and self-management behaviors
Schillinger, Barton, Karter, Wang, & Adler (2006)	Explore the pathway linking HL, education, and glycemic control	395 adults with diabetes recruited from primary care clinics between June and December 2000 in San Francisco, CA Country: United States Mean age: 57.9 y; uninsured: 30.6%; primary English speakers: 51.7%; <HS graduate: 46.8%; Income <$10,000: 68.8% Ethnicity: 18.5% Asian/Pacific Islander, 25.3% Black, 13.9% White, 42.3% Hispanic HL levels: not stated	Functional literacy (s-TOFHLA)	Direct relationship between educational attainment and HL: HS (beta = 0.24, *p*< .05), some college (beta = 0.51, *p*< .05). Direct association between educational attainment and glycemic control: HS (beta = −0.11, *p* < .05), some college (beta = −0.06, *p*< .05). HL mediates relationship between educational attainment (HS education (beta = −0.04, *p <*.05) and some college education (beta = −0.08, *p*< .05) and glycemic control
Soones et al. (2017)	Describe causal pathway linking HL to medication adherence	433 older adults with asthma recruited from hospital and community practices in New York and Chicago Country: United States Age: 60–70 y; mean age: 67 y; female: 84%, <HS graduate: 32.6%; Income <$1,350/month: 54% Ethnicity: 31% Black, 39% Hispanic HL levels: adequate: 64%; limited: 36%	Comprehension and numeracy (s-TOFHLA)	Concerns about medication associated with low HL (beta = −0.154, *p*<.001) and lower medication adherence (beta = −0.2, *p*< .004). Low HL associated with low medication adherence through medication concerns (beta = 0.033, *p*= .002). Direct relationship between HL and medication adherence (beta = 0.123, *p*< .001). Cognition directly associated with HL(beta = −0.767, *p* < .001). Nonsignificant relationships between HL and medication necessity and illness beliefs and medication adherence
Sun et al. (2013)	Develop and validate a HL model to explain the determinants of HL and the associations between HL and health behaviors	3,222 city-dwelling Chinese adult residents Country: China Age: 16–81 y; mean age: 33.8 y; <HS graduate: 38.4%; income <3,000 Yuan (∼$438): 83.2% Ethnicity: 100% Asian HL levels: not stated	Print literacy, numeracy (Skill-based HL tool)^a^	Education has positive and direct effect on prior knowledge of infectious respiratory diseases (beta = 0.324, *p*< .01) and HL (beta = 0.346) HL directly related to health behavior (beta = 0.101). Age directly associated with health status (beta = 0.107)
Zou, Chen, Fang, Zhang, & Fan (2017)	Explore factors associated with self-care behaviors and examine mediating role of self-care confidence	321 adults with chronic heart failure recruited from cardiovascular units in Shandong, China Country: China Mean age: 64 y; female: 49%; <HS graduate: 65.1%; unemployed: 59.2%; income <1,000 Yuan (∼$155): 27.4% Ethnicity: 100% Asian HL levels: not stated	Functional Literacy (Chinese version of Health Literacy Scale for patients with Chronic Disease)	Functional capacity (beta = 0.155, *p*< .01) and knowledge (beta = 0.321, *p*< .01) directly associated with self-care management. HL (beta = 0.043, *p*< .01) and social support (beta = 0.146, *p*< .01) are directly associated with self-care maintenance. Self-care confidence is directly associated with both self-care maintenance (beta = 0.123, *p* < .05) and management (beta = .309, *p*< .01). Age (beta = 0.194, *p*< .01) and health failure duration (beta = 0.105, *p*< .05) are significantly associated with self-care maintenance. Self-care confidence mediates relationships between knowledge (beta = 0.0225, *p*< .01), HL (B = 0.162, *p*< .01), social support (beta = 0.174, *p*< .01), and self-care behaviors

Note. Design of all the studies was cross-sectional except for the study by Intarakamhang & Intarakamhang (2017), which used mixed methods. ED = emergency department; HbA1c = hemoglobin A1C; HL = health literacy; HLS-EU-Q: European Health Literacy Survey Questionnaire; HRQOL = health-related quality of life; HS = high school; REALM = Rapid Estimate of Adult Literacy in Medicine; SES = socioeconomic status; S-TOFHLA = Short Test of Functional Health Literacy in Adults; TOFHLA = Test of Functional Health Literacy in Adults.

a Health literacy instrument designed for purposes of the study.

**Table 3:** Theoretical Frameworks of Health Literacy

**Reference**	**How Framework Was Informed**	**Proposed Antecedents to HL**	**Proposed Mediators and Moderators**	**Hypothesis Tested**	**Health Behaviors/Outcomes**	**Fit Indices for Final Models**
Brega et al. (2012)	Not stated	Age, gender, income, education	Mediators: diabetes knowledge; behavior (healthy and unhealthy food consumption, physical activity, self-monitoring blood glucose) Moderators: none	Diabetes-related knowledge and behavior (healthy diet, physical activity, self-monitoring of blood sugar) mediate relationship between HL and glycemic control	Glycemic control	*X^2^* = 976.78, *df*= 255 (*p* not reported) CFI: 0.85 RMSEA: 0.03 Acceptable fit
Chen et al. (2014)	Orem's theory of self-care; Bandura's social cognitive theory	Years of formal education	Mediators: knowledge; self-efficacy Moderators: none	Formal education is associated with HL and has a direct effect on heart failure knowledge. Direct relationship among HL, health failure knowledge, and self-efficacy. Heart failure knowledge mediates relationship between HL and self-efficacy. Heart failure knowledge and self-efficacy mediate the relationship between HL and self-care	Heart failure self-care (maintenance and management)	*X*^2^ = 3.05, *df* = 4 (*p*= .55) CFI: 1 RMSEA: 0 GFI: 0.98 NFI: 0.95 Good model fit
Cho, Lee, Arozullah, & Crittenden (2008)	Not stated	Gender, race and education	Mediators: disease knowledge; health behavior; preventive care; medication compliance Moderators: none	Mediating factors (disease knowledge, health behavior, preventive care, and compliance with medication) link HL and outcomes (health status, health care, ED visit and hospitalization)	Health status, hospitalization, ED visit	*X*^2^= 15.26, *df*= 13 (*p*= .29) RMSEA: 0 AGFI: 0.91 NFI: 0.99 Adequate fit
Como (2018)	Paasche-Orlow and Wolf causal pathways linking limited health literacy to health outcomes Bandura's self-efficacy theory	Patient demographics (age, education, ethnicity) Social factors (employment, income, language, social support, marital status) Illness severity indicators (number of medications/days, frequency/day)	Mediators: medication adherence; self-efficacy Moderators: none	HL, medication adherence, and self-efficacy are associated with physical health status. Medication adherence mediates the relationship between HL and physical health status. HL, self-efficacy, and medication adherence are associated with mental health status. Medication adherence mediates the relationship between HL and mental health status	Health outcomes (physical health status, mental health status)	Not reported
Crook, Stephens, Pastorek, Mackert, & Donovan (2016)	Theory of diffusion of innovations	Perceived health knowledge, Internet use	Mediators: information overload; attitude toward information Moderators: none	Frequent Internet use is directly related to high HL; higher perceived health knowledge is directly related to frequent Internet use, high HL, positive attitude toward information, and lower perception of information overload Higher HL associated with lower levels of information overload and positive attitudes toward information Perceived level of information overload negatively predicts attitude toward information Intention to share information positively predicts behavioral intentions; attitude toward information positively predicts behavioral intentions and information-sharing intentions Attitude toward information mediates relationship between HL and behavioral intentions, as well as relationship between perceived overload and information-sharing intentions	Behavioral intention, information sharing	*X^2^* = 13.00, *df* = 12 (*p*= .37) RMSEA: 0.02 CFI: 1 TLI: 0.99 SRMR: 0.06 Good model fit
Guo et al. (2014)	Not stated	Age, gender, race, education, income, having a regular dentist	Mediators: patient-dentist communication; dental care patterns Moderators: none	Hypothesis: high HL associated with better patient-dentist communication, and better communication is in turn associated with increased likelihood to seek regular dental care, resulting in better self-rated oral health	Self-rated oral health	*X^2^*= 0.43 (*p*= .51) RMSEA: 0.01 CFI: 0.99 Good model fit
Hickman, Clochesy, & Alaamri (2016)	Integrated model of client health behavior	None	Mediators: quality of provider interaction; perceived communication skills; behavior activation Moderators: none	The association between HL and blood pressure control is mediated by quality of provider interaction, perceived communication skills, and behavioral activation	Blood pressure control	*X^2^* = 1.1, (*p*= .76) CFI: 1 RMSEA: 0 SRMR: 0.03 TLI: 1.1 Excellent fit
Hou et al. (2018)	Integrated model of HL	Age, education, cancer stage, time since diagnosis, marital status, residential area, occupation	Mediators: none Moderators: none	Intercorrelated determinants of HL (age, education, cancer stage, time since diagnosis, marital status, residential area, occupation) predict patients' HL and influence the consequences of HL (participation in decision-making, self-rated health status). There is direct relationship between determinants and consquences of HL	Participation in shared decision-making Self-rated health status	*X^2^* = 55.12, *df* = 32 (*p*= .007) RMSEA: 0.04 CFI: 0.99 SRMR: 0.03 AIC: −8.88 Good model fit
Intarakamhang & Intarakamhang (2017)	Nutbeam model	Health knowledge	Mediators: none Moderators: none	Direct relationship between basic health skill (health knowledge and understanding) and eating behaviors. Association between basic health skill (health knowledge and eating behaviors) is mediated by interactive skills (communicating for added skills) and critical skills (making appropriate health-related decision)	Obesity preventive behaviors (eating behaviors, exercise behaviors, and emotional coping)	*X^2^*= 60.1, *df*= 12 (*p*= .00) RMSEA: 0.05 CFI: 0.99 AGFI: 0.99 PNFI: 0.72 Good model fit
Jin, Lee, & Dia (2019)	HL skills framework, cognitive mediation model	Online information seeking behaviors (using emails to communicate with providers; visit social networking site to read and share medical topics)	Mediators: decisional balance; information overload Moderators: none	Online health information-seeking behavior is positively associated with HL Online health information-seekingbehavior is associated with information overload Information overload is inversely associated with HL HL is positively associated with colorectal cancer screening HL is positively associated with decisional balance Decisional balance is positively associated with colorectal cancer screening	Colorectal cancer screening	Not reported
E.H. Lee, Lee, & Moon (2016)	Not stated	Age, gender, education, marital status, treatment regimen (diet/exercise, insulin, oral hypoglycemic only, oral hypoglycemic & insulin), HbA1c, duration of disease	Mediators: self-efficacy; self-care activities Moderators: none	Study aim: test relationship among HL, self-efficacy, self-care activities, and HRQOL	HRQOL (emotional suffering, social functioning, adherence to treatment, diabetes-specific symptoms)	*X^2^*= 265.79, *df*= 71 RMSEA: 0.07 CFI: 0.92 GFI: 0.92 SRMR: 0.07 NFI: 0.92 Good model fit
Y.J. Lee et al. (2016)	Paasche-Orlow and Wolf model	Education, age, empowerment perceptions	Mediators: self-efficacy; self-care behaviors (medication, exercise, diet, blood sugar monitoring, adversity prevention) Moderators: none	Self-care behaviors mediate relationship between HL and glycemic control (i.e., HbA1c) Direct relationships: (1) HL and self-efficacy, (2) HL and glycemic control; (3) empowerment and HL, self-care behaviors, self-efficacy, and glycemic control	Glycemic control (HbAIc)	*X^2^*/ *df* = 1.79 RMSEA: 0.052 CFI: 0.94 GFI: 0.95 AGFI: 0.96 AIC: 145.25 Acceptable model fit
Osborn, Cavanaugh, et al. (2011)	Not stated	Race	Mediators: none Moderators: none	Black race associated with poor medication adherence; numeracy associated with medication adherence and explains association between race and adherence	Medication adherence	*X^2^*= 0.08 (*p*= 0.78) RMSEA: 0.00 CFI: 1.00 Excellent model fit
Osborn, Cavanaugh, Wallston, & Rothman (2010)	Not stated	None	Mediators: diabetes self-efficacy Moderators: none	HL is directly related to glycemic after controlling for demographics (age, gender, race, education, income, insulin use, diabetes type, and years since diagnosis). Self-efficacy mediates HL and glycemic control	Glycemic control	*X^2^*= 6.17, (*p*= 0.41) CFI: 1 RMSEA: 0.01 Excellent model fit
Osborn, Paasche-Orlow, Bailey, & Wolf (2011)	Paasche-Orlow and Wolf model	Race, education, age	Mediators: knowledge; self-efficacy; self-care Moderators: none	Patient demographics (race/ethnicity, education, age) predict HL HL predicts determinants of self-care at the patient level (knowledge and self-efficacy) Patient-level determinants of self-care predict self-care behavior (physical activity) Self-care behavior predicts health status (subjective health)	Health status (subjective health)	*X^2^*= 6.75, (*p*= .40) RMSEA: 0.01 CFI: 1 Excellent model fit
Photharos, Wacharasin, & Duongpaeng (2018)	Individual and family self-management theory	None	Mediators: chronic kidney disease self-efficacy Moderators: none	Family functioning, illness perception, and HL directly affect self-management behaviors and indirectly affect self-management behaviors through chronic kidney disease self-efficacy Family functioning influences self-management behaviors through social support	Self-management behaviors (adherence to chronic kidney disease recommendation, self-integration, problem solving, seeking social support)	*X^2^*/ *df* = 1.63 RMSEA: 0.48 GFI: 0.93 AGFI: 0.9 Acceptable model fit
Schillinger, Barton, Karter, Wang, & Adler (2006)	Not stated	Educational level, age, primary language, health insurance status	Mediators: none Moderators: none	HL mediates the relationship between education level and glycemic control	Glycemic control	*X^2^*= 12.22, *df* = 31 (*p*= 0.10) RMSEA < 0.0001 CFI: 1 AGFI: 0.99 Good model fit
Soones et al. (2017)	Not stated	Cognition	Mediators: illness beliefs; medication concerns; medication necessity Moderators: none	Asthma illness and medication beliefs mediate the relationship between HL and medication adherence	Medication adherence	RMSEA: 0.05 CFI: 0.93 Adequate fit
Sun et al. (2013)	Baker, Paasche-Orlow	Age, education, income, prior knowledge of infectious respiratory diseases	Mediators: health behavior Moderators: none	Prior knowledge influences development of HL skills HL has direct effect on health behaviors HL mediates relationship between prior knowledge and health behavior Health behavior influences health status	Health status	*X^2^*: 10.22, *df*= 6 (*p*= .1159) RMSEA: 0.05 CFI: 0.1 AGFI: 0.1 Good model fit
Zou, Chen, Fang, Zhang, & Fan (2017)	Capability opportunity motivation and behavior model	None	Mediators: self-care confidence Moderators: None	Capability (functional capacity, knowledge, HL) and opportunity (social support, socioeconomic status) are associated with behavior (self-care maintenance, self-care management) through motivation (self-care confidence)	Heart failure self-care maintenance Heart failure self-care management	*X^2^*= 14.04, *df*= 11 (*p*= .23) RMSEA: 0.029 CFI: 0.99 Good model fit

Note. AGFI: Adjusted Goodness of Fit; AIC: Akaike Information Criterion; CFI = Comparative Fit Index; DF = degrees of freedom; ED = emergency department; GFI = Goodness of Fit Index; HbA1c = hemoglobin A1c; HL = health literacy; HRQOL = health-realted quality of life; NFI = Normed Fit Index; RMSEA = root mean square error of approximation; X^2^ = chi-square.

**Table A:** Database Search Strategy

**PubMed**
((“HL”[Mesh] OR “HL”)) AND (“Models, Theoretical”[Mesh] OR “conceptual framework” OR “conceptual frameworks” OR “conceptual model” OR “conceptual models”)
**CINAHL**
((MH “Conceptual Framework”) OR (“conceptual framework”) OR (conceptual N3 (framework* OR model*)) OR (MH “Models, Theoretical+”) OR (“theoretical models”) AND ((MH “HL”) OR (“HL”) OR (health N3 (literacy OR literate OR illiteracy OR illiterate))
**Embase**
“HL”/exp OR (health NEAR/3 (literacy OR literate OR illiterate OR illiteracy)):ab,ti AND “conceptual framework”/exp OR (conceptual NEAR/3 (framework* OR model*)):ab,ti OR “theoretical model”/exp
